# The Effect of Neighborhood Disadvantage on the Racial Disparity in Ovarian Cancer-Specific Survival in a Large Hospital-Based Study in Cook County, Illinois

**DOI:** 10.3389/fpubh.2015.00008

**Published:** 2015-01-22

**Authors:** Caryn E. Peterson, Garth H. Rauscher, Timothy P. Johnson, Carolyn V. Kirschner, Sally Freels, Richard E. Barrett, Seijeoung Kim, Marian L. Fitzgibbon, Charlotte E. Joslin, Faith G. Davis

**Affiliations:** ^1^Division of Epidemiology and Biostatistics (MC 923), School of Public Health, University of Illinois at Chicago, Chicago, IL, USA; ^2^Survey Research Laboratory, Public Administration, University of Illinois at Chicago, Chicago, IL, USA; ^3^Division of Gynecologic Oncology, NorthShore University HealthSystem, Evanston, IL, USA; ^4^Department of Obstetrics and Gynecology, University of Chicago, Chicago, IL, USA; ^5^Center for Health Behavior Research, University of Illinois at Chicago, Chicago, IL, USA; ^6^Division of Health Policy and Administration, School of Public Health, University of Illinois at Chicago, Chicago, IL, USA; ^7^Department of Medicine, School of Public Health, University of Illinois at Chicago, Chicago, IL, USA; ^8^Department of Ophthalmology and Visual Sciences, University of Illinois at Chicago, Chicago, IL, USA; ^9^School of Public Health, University of Alberta, Edmonton, AB, Canada

**Keywords:** ovarian cancer and socioeconomic status, survival analysis, healthcare disparities, neighborhood effect, racial disparities in ovarian cancer

## Abstract

This paper examines the effect of neighborhood disadvantage on racial disparities in ovarian cancer-specific survival. Despite treatment advances for ovarian cancer, survival remains shorter for African-American compared to White women. Neighborhood disadvantage is implicated in racial disparities across a variety of health outcomes and may contribute to racial disparities in ovarian cancer-specific survival. Data were obtained from 581 women (100 African-American and 481 White) diagnosed with epithelial ovarian cancer between June 1, 1994, and December 31, 1998 in Cook County, IL, USA, which includes the city of Chicago. Neighborhood disadvantage score at the time of diagnosis was calculated for each woman based on Browning and Cagney’s index of concentrated disadvantage. Cox proportional hazard models measured the association of self-identified African-American race with ovarian cancer-specific survival after adjusting for age, tumor characteristics, surgical debulking, and neighborhood disadvantage. There was a statistically significant negative association (−0.645) between ovarian cancer-specific survival and neighborhood disadvantage (*p* = 0.008). After adjusting for age and tumor characteristics, African-American women were more likely than Whites to die of ovarian cancer (HR = 1.59, *p* = 0.003). After accounting for neighborhood disadvantage, this risk was attenuated (HR = 1.32, *p* = 0.10). These findings demonstrate that neighborhood disadvantage is associated with ovarian cancer-specific survival and may contribute to the racial disparity in survival.

## Introduction

Ovarian cancer is the fifth leading cause of cancer death among U.S. women, with an estimated 21,980 new cases diagnosed in 2014 and 14,270 deaths (1). Across study populations, the incidence of ovarian cancer has been consistently higher in White women than in African-American women (2–4). Paradoxically, African-Americans have been consistently found to have poorer survival than Whites at all stages of this disease (5–7). Recent SEER data show improvements in the 5-year relative survival for women diagnosed with ovarian cancer, yet these rates remain significantly lower for African-American women compared to Whites (36 and 44%, respectively) (1).

Racial disparities in cancer survival are typically assessed solely in terms of individual-level factors, and rightly so. Racial differences in individual-level demographic and clinical factors play an important role in prolonging or shortening survival following diagnosis of various types of cancer (8–10). Yet, these individual-level differences may be due in part to differences in neighborhood environments, and excluding this important contextual factor may produce an incomplete picture. This analysis explores the role that neighborhood, and in particular neighborhood disadvantage, plays in explaining the racial disparity in ovarian cancer survival.

Sampson and colleagues (11, 12) have defined neighborhoods as “ecological units nested within successively larger communities.” Implicit in this definition is the concept of neighborhood differentiation, which includes aspects such as social inequality between neighborhoods and the idea that neighborhood characteristics can influence aspects of residents’ lives. One aspect of social inequality at the neighborhood level is residential segregation. Massey and Denton (13) describe the damaging social consequences of residential segregation, which include social and economic isolation as well as structural environments characterized by physical decay, crime, and social disorder. Ellen and colleagues (14) propose several pathways by which neighborhoods can affect health. For example, weak neighborhood resources can reduce access to and quality of healthcare, and physical stresses in a neighborhood can pose challenges to health-promoting behaviors such as physical activity and healthy eating.

Neighborhood disadvantage has been implicated in racial disparities across a variety of health outcomes (15–17) above and beyond individual-level demographic, socioeconomic, and clinical factors (18–20), and differences in neighborhood disadvantage may also contribute to disparities in ovarian cancer-specific survival. Sampson and colleagues (12) suggest several ways in which the effect of neighborhood disadvantage may be particularly strong for African-Americans. These include the connection between concentrated disadvantage and residential isolation, as well as the “bundling” of social problems that occur at the neighborhood level, such as weak bonds of social support, economic uncertainty, and both social and physical disorder. Residence in high-poverty neighborhoods has been associated with shorter survival in individuals diagnosed with breast cancer (21, 22), prostate cancer (23), and lung cancer (24), and this may also be the case for women with ovarian cancer.

Using Browning and Cagney’s (25) index of concentrated disadvantage, we examined whether neighborhood disadvantage was associated with cancer-specific survival, and whether this association helped to explain any observed survival disparity among African-American and White women diagnosed with ovarian cancer in Cook County, IL, USA, which includes the city of Chicago.

## Materials and Methods

### Study population

Cases from an original case–control study examining the etiology of ovarian cancer were recruited from hospitals in Cook County, IL, USA between June 1, 1994, and December 31, 1998. Eighty-three percent of hospitals in Cook County participated in the study. These hospitals represent a wide array of community hospitals in the Chicago metropolitan area, including major teaching hospitals, county hospitals, and system affiliated hospitals in a range of sizes. Identified cases were subsequently sent to the Illinois State Cancer Registry (ISCR) to determine whether any eligible cases had been missed. Of the 1,562 identified by ISCR during the ascertainment period, 1,210 (77.5%) were part of the original case–control study. The remaining 352 ISCR cases were excluded for the following reasons: cases could not be reviewed for eligibility because they were diagnosed in a non-participating hospital (*n* = 130), the medical record could not be obtained (*n* = 75), the case was identified through death certificate (*n* = 35), and the case did not meet the study’s age or race eligibility criteria (*n* = 112). Diagnosis was confirmed after surgical biopsy using the International Histological Classification of Ovarian Tumors recommended by the Féderation Internationàle de Gynécologie et d’Obstétrique (FIGO) (26). A pathology review of 386 of the eligible cases was conducted by an independent gynecologic pathologist. Hospital pathology reports were used for histologic classification for the remaining cases.

Cases of epithelial ovarian cancer (“ovarian cancer”) were eligible for inclusion in this analysis if they were residents of Cook County, treated at one of the participating hospitals, 18–74 years old at the time of diagnosis, and self-reported their race as either “Black” or “White.” Among the 1,210 cases available between June 1, 1994 and December 31, 1998, 702 met the eligibility criteria. After reviewing histology codes, 102 tumors were determined to be either benign, stromal, or of germ-cell origin, and were subsequently excluded from this analysis. Of the remaining 600 cases, vital status was valid for 581 women. The protocol was approved by the University of Illinois at Chicago Institutional Review Board.

### Vital status

Case information was submitted to the National Death Index (NDI) and matched through December 2008. Vital status was determined through a manual review of the NDI Summary file, with dates of death recorded for all linked cases. In cases with more than one possible match, the record with the most data items in agreement was used. Unlinked cases were right-censored at the last date of December 31, 2008. The cause of death was classified as either ovarian cancer or ovarian cancer-related death using the underlying and selected-cause codes from the appropriate revision of the International Classification of Diseases (ICD-9, -10). The study endpoint was ovarian cancer-specific survival time calculated by subtracting the date of ovarian cancer diagnosis from the date of death due to ovarian cancer or censoring at the last date of December 31, 2008.

### Variables

A composite variable representing neighborhood disadvantage was constructed using U.S. Census data and was based on Browning and Cagney’s *concentrated disadvantage* factor, which was dominated by high factor loadings for percent below the poverty line, unemployed, in female-headed households, under age 18, and African-American (25). Because the case-ascertainment period (i.e., 1994–1998) spanned two U.S. Census periods, both the 1990 and 2000 census periods were used to develop the variable. Each patient’s residential address at the time of diagnosis was geocoded to the block level and then located within a census tract. Data from the 1990 and 2000 Census periods were used to create interpolated values representing the midpoint in the ascertainment period (i.e., 1996) for each of the following census variables: percent below poverty, percent unemployed, percent receiving public assistance, percent in female-headed households, percent under age 18, and percent African-American. Each interpolated value was standardized (i.e., converted to *z*-scores). The variables were then summed with equal weighting and standardized to create the final disadvantage index variable. Higher scores represented greater concentrated disadvantage.

Stage at diagnosis was analyzed as late-stage (FIGO III/IV) versus early-stage (FIGO I/II) diagnosis. The pathologic grade of tumors was classified as high pathologic grade (moderately to poorly differentiated) versus low grade (well differentiated). The FIGO version of the World Health Organization’s histologic typology of ovarian tumors was used to classify the six categories of epithelial tumors: serous, mucinous, clear cell, endometrioid, undifferentiated, and unclassified. (Tumors were considered unclassified if they could not be assigned to any of the other five groups or if they had no histology.) Epithelial histologic sub-type was analyzed as serous versus all others due to small cell sizes in the non-serous sub-types. Finally, tumors were considered suboptimally debulked following initial surgery when residual lesions were >2 cm (the definition at the time cases were diagnosed), and considered optimally debulked when lesions were 2 cm or less. Only epithelial tumors were included in this analysis.

### Statistical analysis

Differences in the distribution of tumor characteristics by race and quartile of disadvantage were tested using Chi-square and *t*-test statistics for categorical and continuous variables, respectively. Simple linear regression was used to evaluate the association between cancer-specific survival time and disadvantage. The Cochran–Armitage test was used to evaluate the linear trend of variables by quartile of disadvantage. Unadjusted Kaplan–Meier 5- and 10-year survival rates and 95% confidence intervals were estimated for African-American and White women.

Three Cox proportional hazard (PH) models measured the association of race (African-American) with ovarian cancer-specific survival time: Model 1 estimated the age-adjusted hazard of death by race; Model 2 estimated the hazard of death by race controlling for age, tumor characteristics, and surgical debulking; Model 3 estimated the hazard of death by race controlling for age, tumor characteristics, surgical debulking, and neighborhood disadvantage (comparing the highest quartile of disadvantage to the lower three quartiles as the reference category). Product terms tested the interaction between race and disadvantage. No violations of the PHs assumption were observed (*p* values for interaction with time ranged from 0.45 to 0.88). Similar Cox models were run with the shared frailty model to account for clustered data (i.e., cases within census tracts.) Analyses were performed using SAS (v9.3, Cary, NC, USA).

## Results

By the end of the follow-up period, 87 of 100 (87%) African-Americans versus 358 of 481 (74%) Whites had died (*p* = 0.007). The mean survival time for African-Americans was 16.9 months shorter than for Whites (61.5 versus 78.4 months, *p* = 0.007). There was a statistically significant negative association (−0.645) between ovarian cancer-specific survival and neighborhood disadvantage (*p* = 0.008) (Results not shown).

Table 1 presents the distribution and association of patient characteristics by race and quartile of disadvantage. Median survival was 3.18 years for African-Americans versus 5.31 years for Whites (*p* = 0.007). There were no statistically significant differences in tumor characteristics between the two groups of women. African-American women in the study lived in neighborhoods with significantly higher mean disadvantage scores compared to White women (0.79 versus −0.65, *p* < 0.0001), and the majority of African-Americans lived in neighborhoods in the highest quartile of disadvantage (85%), compared to 12% of Whites (*p* < 0.0001).

**Table 1:** **Percent distribution and association of patient characteristics, by race and quartile^a^ of disadvantage (*n* = 581)**.

	Race			By quartile of disadvantage score				
	African-Americans	Whites	*p*	1st	2nd	3rd	4th	*p*_Trend_
	
	*n* = 100 (%)	*n* = 481(%)		*n* = 146 (%)	*n* = 145 (%)	*n* = 145 (%)	*n* = 145 (%)	
Mean survival in years	5.12	6.54	0.007	6.55	6.82	6.45	5.35	0.46
(SD)	(−0.45)	(−0.2)		(−4.8)	(−4.8)	(−4.7)	(−4.7)	
[Median]	[3.18]	[5.31]		[5.27]	[5.61]	[5.30]	[2.99]	
FIGO stage at diagnosis
Early (FIGO I/II)	42 (42)	224 (46.6)	0.40	75 (51.4)	64 (44.1)	69 (47.6)	58 (40)	0.09
Late (FIGO III/IV)	58 (58)	257 (53.4)		71 (48.6)	81 (55.9)	76 (52.4)	87 (60)	
Pathologic grade
Low-grade	26 (26)	150 (31.2)	0.30	48 (32.9)	49 (33.8)	48 (33.1)	31 (21.4)	0.04
High-grade	74 (74)	331 (68.8)		98 (67.1)	96 (66.2)	97 (66.9)	114 (78.6)	
Histologic sub-type
Serous	53 (53)	230 (47.8)	0.35	67 (45.9)	71 (49)	73 (50.3)	72 (49.7)	0.49
All others	47 (47)	251 (52.2)		79 (54.1)	74 (51)	72 (49.7)	73 (50.3)	
Surgical debulking (missing = 25)
Optimal debulking	45 (51.7)	278 (59.3)	0.19	92 (63.9)	82 (59)	82 (58.2)	67 (50.8)	0.03
Suboptimal debulking	42 (48.3)	191 (40.7)		52 (36.1)	57 (41)	59 (41.8)	65 (49.2)	
Mean disadvantage score^b^	0.79	−0.65	<0.0001				
(SD)	(0.84)	(0.34)					
[Range]	[−0.93, 2.33]	[−1.06, 1.71]					
Quartile of disadvantage
Highest (4th Q)	85 (85)	60 (12)	<0.0001				
1st–3rd Quartiles	15 (15)	421 (88)					

*^a^Neighborhood disadvantage divided into fourths at the quartiles of the sample distribution*.

*^b^Higher scores reflect greater concentrated disadvantage*.

The highest quartile of neighborhood disadvantage was associated with late-stage diagnosis (*p* = 0.05), high-grade tumors (*p* = 0.03), suboptimal debulking (*p* = 0.03), and race (*p* < 0.0001). The likelihood of being diagnosed with higher grade tumors and receiving suboptimal debulking was also associated with increasing quartile of disadvantage, and was marginally associated with late-stage diagnosis (*p* = 0.04, *p* = 0.03, and *p* = 0.09, respectively). Because the strongest effect of disadvantage was observed in the highest quartile, this variable was evaluated as a binary variable in the subsequent series of Cox models.

The unadjusted Kaplan–Meier 5-year cancer-specific survival rates for African-Americans and Whites were 41 and 62%, respectively, and 24 and 43%, respectively, for 10-year survival (Table 2).

**Table 2:** **Kaplan–Meier ovarian cancer-specific survival rates (95% CI), by race (*n* = 581)**.

	Survival rate (95% CI)	
	5-year	10-year
African-Americans	0.41 (0.31, 0.51)	0.24 (0.16, 0.33)
Whites	0.62 (0.57, 0.66)	0.43 (0.39, 0.48)

The results of age-adjusted Cox PH models estimating the association between race (African-American) and ovarian cancer-specific mortality are presented in Table 3. African-American women were more likely than White women to die of ovarian cancer (Model 1: HR = 1.54, *p* = 0.004). This increased risk held after adjusting for tumor characteristics (Model 2: HR = 1.59, *p* = 0.003). However, after accounting for neighborhood disadvantage, this risk was attenuated (Model 3: HR = 1.32, *p* = 0.10). There was no statistically significant interaction between race and disadvantage, and a model accounting for clustered data produced nearly identical results to the Table 3 model ignoring clustering (data not shown).

**Table 3:** **Hazard ratios (HR) for African-American versus White ovarian cancer-specific mortality**.

Variables	Model 1^a^		Model 2^b^		Model 3^c^	
	HR (95% CI)	*p*	HR (95% CI)	*p*	HR (95% CI)	*p*
Race
White	1.00 (reference)	0.004	1.00 (reference)	0.003	1.00 (reference)	0.10
African-American	1.54 (1.21, 1.94)		1.59 (1.23, 2.04)		1.32 (0.95, 1.84)	
Stage at diagnosis
Early (FIGO I/II)			1.00 (reference)	0.0012	1.00 (reference)	0.0016
Late (FIGO III/IV)			1.72 (1.24, 2.39)		1.70 (1.22, 2.36)	
Pathologic grade
Low-grade			1.00 (reference)	0.47	1.00 (reference)	0.54
High-grade			1.09 (0.86, 1.39)		1.08 (0.85, 1.37)	
Histologic sub-type
All others			1.00 (reference)	0.48	1.00 (reference)	0.51
Serous			1.08 (0.87, 1.33)		1.07 (0.87, 1.32)	
Surgical debulking (missing = 25)
Optimal debulking			1.00 (reference)	<0.0001	1.00 (reference)	<0.0001
Suboptimal debulking			2.08 (1.52, 2.83)		2.12 (1.55, 2.90)	
Disadvantage
1st–3rd Quartiles combined					1.00 (reference)	0.09
4th Quartile (highest)					1.28 (0.96, 1.70)	

*^a^Adjusted for age at diagnosis*.

*^b^Adjusted for age, stage at diagnosis, pathologic grade, histologic sub-type, and surgical debulking*.

*^c^Adjusted for age, stage at diagnosis, pathologic grade, histologic sub-type, surgical debulking, and concentrated disadvantage*.

## Discussion

Our findings are consistent with similar analyses examining the effect of neighborhood environment on survival in residents with breast (27), prostate (23), and lung cancer (24). To our knowledge there are no published reports examining the effect of neighborhood disadvantage (or neighborhood-level socioeconomic status) on racial disparities in ovarian cancer-specific survival. However, at the individual-level, lower socioeconomic status has been associated with poorer survival in women diagnosed with invasive ovarian cancer (28–30).

This study shows that neighborhood disadvantage is associated with ovarian cancer-specific survival and may contribute to the racial disparity in such survival. Although the pathways through which neighborhood disadvantage influences survival are challenging to measure, we hypothesize several causal relations between indicators of neighborhood disadvantage and ovarian cancer-specific survival. Neighborhood disadvantage adversely affects both individual- and neighborhood-level socioeconomic status, as well as the physical and structural environment of the community (31). At the individual level, poverty may affect survival through higher rates of comorbidities (32), which may influence both physicians’ recommendations and women’s abilities to receive and complete treatment for advanced ovarian cancer (33, 34). Poverty and unemployment are associated with inadequate health insurance (32, 35), which impacts the quality of available healthcare (36–40) in terms of treatment (41) as well as management of treatment and disease complications (42).

In addition, neighborhood socioeconomic status is an important contextual factor that negatively impacts health outcomes beyond an individual’s own circumstances (43, 44). An environment of high poverty, high unemployment, and low educational attainment can produce conditions in which priorities such as personal safety, maintaining employment, and caring for one’s family take precedence over health (45–47). These competing priorities can result in later-stage diagnosis and can make treatment completion difficult (48, 49).

Finally, neighborhoods of high concentrated disadvantage expose residents to environmental stressors including crime, neighborhood disorder and decay, and discrimination (50, 51). These environmental stressors may place residents at risk for high levels of stress and distress (52, 53), both of which have an adverse effect on immune processes involved in cancer progression (54) and may increase the likelihood of advanced ovarian cancer (55, 56). For all of these reasons, living in a neighborhood with increased concentrated disadvantage adds an “additional layer of vulnerability” over and above an individual’s personal circumstances (57).

Although this study provides useful insights into the possible causes of racial disparity in ovarian cancer-specific survival between African-American and White women, we acknowledge important limitations which should be addressed in future research. First, direct measures of healthcare access and utilization were not included in this study, and they should be examined in order to assess their possible contribution to disparate survival. Second, the length of residency within a census tract was not known, so models could not account for potential changes in residency after diagnosis. Third, the impacts of prescribed treatment and patients’ responses to that treatment on the survival disparity were not measured. Research has shown that African-Americans are less likely to receive the type of optimal treatment associated with longer survival (29, 58, 59). It is possible that the African-American women in our study may have received less optimal treatment than Whites in terms of cytoreductive surgery or chemotherapeutic regimen, or both, possibly due to differences in healthcare providers or comorbidities that prevented the use of certain therapies (60). Finally, cases were among women diagnosed with ovarian cancer exclusively in Cook County, IL, USA and from a subset of hospitals within the county, which may not be representative of the general population of ovarian cancer patients in the U.S., particularly with respect to stage at diagnosis or receipt of surgical treatment.

This analysis demonstrates that neighborhood disadvantage is an independent predictor of ovarian cancer-specific survival and may contribute to the racial disparity in survival. This association is most evident in the highest quartile of concentrated disadvantage, suggesting a gradient effect (61) of neighborhood disadvantage on racially disparate survival. Future studies of the racial disparity in ovarian cancer-specific survival should also examine the role of neighborhood disadvantage, accounting for racial differences in treatment.

## Conflict of Interest Statement

The authors declare that the research was conducted in the absence of any commercial or financial relationships that could be construed as a potential conflict of interest.
